# Identification of core genes and pathways between geriatric multimorbidity and renal insufficiency: potential therapeutic agents discovered using bioinformatics analysis

**DOI:** 10.1186/s12920-022-01370-1

**Published:** 2022-10-08

**Authors:** Lingyun Zhang, Jiasheng Cai, Jing Xiao, Zhibin Ye

**Affiliations:** 1 Department of Nephrology, Huadong Hospital Affiliated to Fudan University, No. 221 West Yan’an Road, Shanghai, 200040 People’s Republic of China; 2 Department of Cardiology, Huadong Hospital Affiliated to Fudan University, No. 221 West Yan’an Road, Shanghai, 200040 People’s Republic of China; 3 Shanghai Key Laboratory of Clinical Geriatric Medicine, No. 221 West Yan’an Road, Shanghai, 200040 People’s Republic of China

**Keywords:** Geriatric, Multimorbidity, Renal insufficiency, Text mining, Drug discovery

## Abstract

**Background:**

Geriatric people are prone to suffer from multiple chronic diseases, which can directly or indirectly affect renal function. Through bioinformatics analysis, this study aimed to identify key genes and pathways associated with renal insufficiency in patients with geriatric multimorbidity and explore potential drugs against renal insufficiency.

**Methods:**

The text mining tool Pubmed2Ensembl was used to detect genes associated with the keywords including "Geriatric", "Multimorbidity" and "Renal insufficiency". The GeneCodis program was used to specify Gene Ontology (GO) biological process terms and Kyoto Encyclopedia of Genes and Genomes (KEGG) pathways. Protein–protein interaction (PPI) networks were constructed using STRING and visualized in Cytoscape. Module analysis was performed using CytoHubba and Molecular Complex Detection (MCODE) plugins. GO and KEGG analysis of gene modules was performed using the Database for Annotation, Visualization and Integrated Discover (DAVID) platform database. Genes clustered in salient modules were selected as core genes. Then, the functions and pathways of core genes were visualized using ClueGO and CluePedia. Finally, the drug-gene interaction database was used to explore drug-gene interactions of the core genes to identify drug candidates for renal insufficiency in patients with geriatric multimorbidity.

**Results:**

Through text mining, 351 genes associated with "Geriatric", "Multimorbidity" and "Renal insufficiency" were identified. A PPI network consisting of 216 nodes and 1087 edges was constructed and CytoHubba was used to sequence the genes. Five gene modules were obtained by MCODE analysis. The 26 genes clustered in module1 were selected as core candidate genes primarily associated with renal insufficiency in patients with geriatric multimorbidity. The HIF-1, PI3K-Akt, MAPK, Rap1, and FoxO signaling pathways were enriched. We found that 21 of the 26 selected genes could be targeted by 34 existing drugs.

**Conclusion:**

This study indicated that *CST3*, *SERPINA1*, *FN1*, *PF4*, *IGF1*, *KNG1*, *IL6*, *VEGFA*, *ALB*, *TIMP1*, *TGFB1*, *HGF*, *SERPINE1*, *APOA1*, *APOB*, *FGF23*, *EGF*, *APOE*, *VWF*, *TF*, *CP*, *GAS6*, *APP*, *IGFBP3*, *P4HB,* and *SPP1* were key genes potentially involved with renal insufficiency in patients with geriatric multimorbidity. In addition, 34 drugs were identified as potential agents for the treatment and management of renal insufficiency.

## Introduction

With the rapid progress of global aging, the proportion of the geriatric population is gradually increasing, and the growth rate of the population above 80 years old is much higher than that of the population over 65 years old [1]. Geriatric people are prone to suffer from multiple chronic diseases, also known as multimorbidity. The latter refers to the state of having two or more chronic diseases simultaneously [2].

Related studies have revealed that the incidence of multimorbidity in the geriatric population ranges from 55 to 98% [3]. The incidence of renal disease in the geriatric population is much higher than that in the middle and young populations, and the prognosis for the geriatric population is relatively poor [4]. Chronic kidney disease (CKD) generally occurs in the spectrum of geriatric multimorbidity and there are many chronic diseases co-existing with CKD, which are mostly classified as consistent/inconsistent multimorbidity with CKD [5]. Using a single creatinine-based estimating glomerular filtration rate (eGFR) calculation for physiological changes in the body composition that occur with aging does not apply to all adult groups [6]. In the geriatric population, age-related physiological renal changes may lead to a decrease in eGFR. In addition, polypharmacy is common among patients with geriatric multimorbidity due to the presence of multiple diseases. In western countries, the rate of polypharmacy in geriatric multimorbidity patients over 65 years old can reach 30–40% [7]. Therefore, it is imperative to accurately evaluate renal function and identify the pathogenesis of renal insufficiency in geriatric multimorbidity patients to simplify treatment regimens, adjust the dosage and rationalize medication use. Old age is also a factor that interacts with metabolic diseases such as hypertension and diabetes. Therefore, it is critical to evaluate whether renal insufficiency in patients with geriatric multimorbidity is caused by diseases or old age.

Current eGFR assessments in the elderly are mainly derived from equations used to assess eGFR in the young. The 2012 Kidney Disease: Improving Global Outcomes (KDIGO) Clinical Practice Guidelines recommends using the creatinine-derived Chronic Kidney Disease Epidemiology Collaboration (CKD-EPI) equation to estimate eGFR in routine practice. The guidelines suggest that cystatin C-based equations have the potential to improve the diagnosis and epidemiology of CKD and should only be used in individuals with an eGFR between 45 and 59 ml/min/1.73 m^2^ with no other evidence of CKD [8], suggests limited availability of eGFR assessments rather than poor performance. Assessing the eGFR by measuring biomarkers in urine or blood is invasive and time-consuming. There has been significant progress in using transdermal measurements of eGFR in murine models [9, 10], which are non-invasive and allow real-time measurement of eGFR. Transdermal measurement of eGFR may be the key to the non-invasive and real-time measurement of eGFR in various clinical settings in the future.

At present, multiple studies have established that the interaction between metabolic diseases and aging affects renal function in older people and the underlying pathogenic cause is cellular dysfunction. A meta-analysis of genome-wide association studies found that the genes identified in the eGFR locus are highly expressed in renal tissues and pathways associated with renal development, transmembrane transporter activity, renal structure, and the regulation of glucose metabolism [11]. Metabolic analysis in CKD animal models also revealed alterations in numerous metabolites such as metabolic reprogramming of the S-nitroso-coa reductase system that can prevent renal insufficiency. The regulation of *PKM2* in the mouse proximal tubules may be a new perspective in the treatment of renal insufficiency [12]. At the cellular level, it has also been observed that blood glucose and albumin affect the cellular metabolic expression of four kinds of innate cells in the kidneys [13]. For patients with moderate to high risk of CKD, bariatric surgery can maintain better levels of eGFR in obese patients, suggesting that the incidence of renal disease can be reduced by improving metabolic risk [14]. It has also been determined that blood homocysteine level in the elders is closely related to age-induced renal insufficiency [15], suggesting that renal insufficiency in older people is related to cell metabolism.

Geriatric multimorbidity complicated with renal insufficiency can influence the prognosis of patients. However, most of the above studies have assessed the risk of renal disease development in a certain disease, and there is still a lack of tools to accurately assess the impact of multimorbidity on renal insufficiency. Clinicians should precisely identify renal insufficiency in geriatric multimorbidity patients to provide an early diagnosis and treatment to slow down the progression and prevent or delay end-stage renal disease (ESRD). Discovering novel pharmacotherapy by traditional means can be time-consuming and expensive, while treating diseases beyond their original development purpose through drug reuse may be more effective and faster [16]. New information about old drugs and new therapies can be obtained through the text mining of bioinformatics [17]. The purpose of this study was to explore the existing published literature and biological databases and use other analysis tools for the assessment of renal insufficiency in geriatric multimorbidity patients, to further clarify the molecular mechanism of renal insufficiency in geriatric patients with multimorbidity and identify potential therapeutic targets to guide rational clinical drug use better.

## Methods and materials

### Text mining

Text mining was conducted using Pubmed2Ensembl (http://www.pubmed2ensembl.org), which is an extension of the BioMart system. It links more than 2 million articles in PubMed to approximately 150,000 genes from 50 species in Ensembl [18]. We entered "Geriatric" and "Multimorbidity" in the search box in Pubmed2Ensembl. Then non-duplicate genes were extracted. The union of the extracted genes from the two gene sets was defined as the gene set associated with "Geriatric Multimorbidity" and was denoted as the "G-M" gene set. We entered the search term "Renal insufficiency " in Pubmed2Ensembl to extract the unrepeated genes. After retrieving the related genes, the "G-M" and "Renal insufficiency" genes sets were intersected using Venny (https://bioinfogp.cnb.csic.es/tools/venny/). The intersection of genes extracted from two gene sets constituted our text mining genes (TMGs).

### Biological process and pathway enrichment analysis of TMGs

Genecodis is a powerful web-based tool for the functional interpretation of genomics experimental technical results, which integrates different sources of information to search for annotations that often coexist in a set of genes and ranks them based on statistical significance [19]. The TMGs from our text mining were entered and analyzed using the GO biological process analogy. Then, significantly enriched biological process genes were selected. The enriched genes were further analyzed using the KEGG pathways annotation. Afterward, the genes involved in the significantly enriched KEGG pathways were selected for further analysis.

### Integration of protein–protein interaction (PPI) network and identification of Hub genes

Results obtained from the gene enrichment analysis in the previous step were applied to the gene retrieval tool STRING (http://string-db.org) for PPI analysis [20]. STRING provides a platform that can analyze PubMed text mining data and integrate multiple database resources. It covers about 24.6 million proteins from 5090 organisms and more than 3.1 billion interactions, which can be used to analyze the relationships and interactions between proteins. First, STRING was employed to construct the PPI network of different genes. In addition, the application of CytoHubba in Cytoscape was used to identify hub genes [21]. CytoHubba for hub Genes is a Cytoscape plugin that provides 11 topological analysis methods to sort nodes in a network based on the network characteristics [22]. According to the results of correlation analysis, the relevant genes with a degree ≥ 10 were screened out for further analysis.

### Molecular Complex Detection (MCODE) analysis of subnet networks

MCODE constructed by Cytoscape was downloaded and ran using a Cytoscape visualization network of molecular interaction to screen important gene modules in the visual network of molecular interaction [23]. In the analysis results, the gene modules in the analysis were sorted according to the network score. The gene module with the highest score was an important gene module in the visual network of molecular interaction screening, which represented the most critical and typical genes in the network. Two gene modules (including 40 genes) with the highest network score from the PPI network were selected for further verification and analysis.

### Gene ontology and KEGG pathway enrichment analysis of module genes

The GO functional and KEGG pathway enrichment analysis of important module genes was performed using the online gene function analysis tool DAVID (https://david.ncifcrf.gov/) [24]. DAVID is a database of annotation, visualization and integrated discovery of biological information, which can associate the genes in the input list to the term of biological annotation and find the most significantly enriched biological annotation through statistical methods. At present, it is mainly used for related function and pathway enrichment analysis of differential genes. The FDR we chose in the DAVID analysis uses the Benjamin-Hochberg multiple test method to approximate control the error detection rate [25]. Before Gene ontology and KEGG pathway enrichment analysis, we set FDR < 0.05 was the cut-off point. GO (http://www.geneontology.org) database contains three main categories of genome data function classification terms including biological process, cell composition and molecular work [26]. KEGG (www.genome.jp/kegg) is a knowledge base for systematic analysis, annotation and visualization of gene function [27]. We set the Cytoscape plugins ClueGo and Cluepedia to *P* < 0.01 was considered statistically significant to visualize the GO function of core genes and KEGG pathway enrichment analysis [28].

### Drug-gene interactions

The drug-gene interaction database (DGIDB) mines available resources to generate hypotheses about how mutated genes can be targeted for therapy or prioritized for drug development [29]. It provides information on drug-gene interactions and known or potential drug associations to genes. DGIDB was utilized to explore drug-gene interactions associated with the identified genes to generate potential targets for existing drugs or compounds.

## Results

### Acquisition of TMGs

**Fig. 1:**
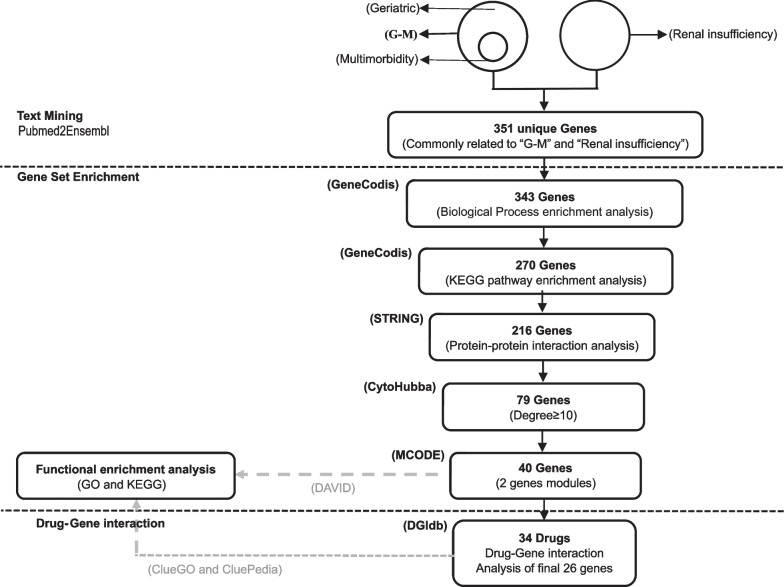
Summary of the whole study design. Text mining procedures were conducted using Pubmed2Ensembl to identify genes related to Geriatric Multimorbidity (G-M) and Renal insufficiency. Genes collection enrichment was used GeneCodis to find genes enriched in the GO biological process terms and KEGG pathways. STRING and CytoHubba were used to construct a protein–protein interaction network and screen the proteins encoded by the hub genes according to the degree of the nodes. MCODE were used to identify the related protein network modules and calculate the score of each module. DAVID and ClueGO were used to analyze the GO biological process terms and KEGG pathways. DGIDB was used to classify potential drug targets based on the lists of significant genes. KEGG: Kyoto Encyclopedia of Genes and Genomes

**Fig. 2:**
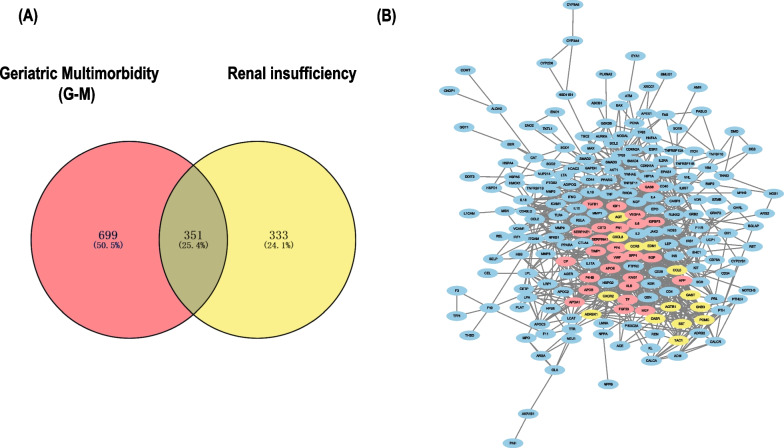
Identification and enrichment analysis of the TMGs. **A** Venn diagram analysis was carried out between the G-M and Renal insufficiency using the Venny website. The 351 genes that were common were considered to be associated to G-M and Renal insufficiency. **B** The protein–protein interaction (PPI) network of the 216 target TMGs were visualized by Cytoscape

Based on the text mining strategy described in the Methods and Materials section (Fig. 1), 1046 unique genes were related to the geriatric population, 18 unique genes were related to multimorbidity, and 684 unique genes were related to renal insufficiency. Then, all non-duplicate genes were extracted. The union of the extracted genes from the two gene sets associated with geriatric multimorbidity was denoted as the G-M gene set. Among them, 351 genes overlapped between the G-M and the renal insufficiency set. Hence, we considered that those 351 genes participated in the biological processes of renal insufficiency in patients with geriatric multimorbidity (Fig. 2A).

### Gene ontology and KEGG pathway analysis

The GeneCodis website was used to visualize GO functional and KEGG pathways to determine the most enriched terms closely related to renal insufficiency in patients with geriatric multimorbidity. The result of our analysis showed that 2717 significantly enriched GO biological process annotations were identified as 343 unique genes. Among them, the five most enriched terms were "cytokine-mediated signaling pathway" (*P* = 2.02245E−57), "signal transduction" (*P* = 1.40787E−45), "negative regulation of apoptotic process" (*P* = 5.31297E−44), "positive regulation of gene expression" (*P* = 4.56741E−41) and "response to drug" (*P* = 5.19875E−39), for 52, 77, 51, 48 and 39 TMGs, respectively (Table 1). Other highly enriched biological processes included "positive regulation of transcription by RNA polymerase II", "inflammatory response", "response to lipopolysaccharide", "cellular protein metabolic process" and "response to hypoxia".

**Table 1:** Top 10 enriched GO terms assigned to the text mining genes

Process	Genes in query set	Total genes in genome	Corrected hypergeometric *P* value	Genes
Cytokine-mediated signaling pathway	52	298	2.02245E−57	CDKN1A, CD4, GRAP2, CASP3, TNFSF11, VIM, VEGFA, VCAM1, TP53, TNFRSF1B, TNF, TIMP1, TGFB1, SHC1, CCL5, CCL2, SAA1, BCL2, RELA, PTGS2, PRTN3, POMC, PF4, MUC1, MMP9, MMP3, MMP2, MMP1, KIT, JAK2, ITGAM, FASLG, IL18, IL17A, IL10, IL8, IL6ST, IL6, IL4, IL2RA, IL2, IL1B, IFNA1, ICAM1, HMOX1, HIF1A, HGF, GRB2, FN1, AKT1, F3, CCR5
Signal transduction	77	1541	1.40787E−45	CD34, CD4, ADIPOQ, CASR, TNFRSF10A, TNFSF10, CALCR, VEGFA, SCGB1A1, TTR, TLR4, TIMP1, TIE1, SRI, SPP1, SOX9, SHC1, CCL5, CCL2, BCR, OPN1SW, TNFRSF17, S100A6, RET, PTHLH, PRL, PPARG, POMC, PLXNA2, ATM, TNFRSF11B, NGF, NFKB1, NR3C2, MAS1, LTA, LEP, KIT, JAK2, FASLG, FAS, IRS1, INS, IL18, IL10, IL8RB, IL8, IL6ST, IL6, IL4, IL1B, IGFBP2, IGFBP1, IGF1, IFNA1, ANXA5, HIF1A, GSK3B, ABR, GNB3, GH1, GAS6, GAST, FLNB, AKT1, ESR1, EPO, EPAS1, AGTR1, EGF, RAPH1, RETN, ADRBK1, ADRB2, CD244, ADM, CCR5
Negative regulation of apoptotic process	51	509	5.31297E−44	CDKN1A, CD44, CD40LG, CD28, CAT, CASP3, WT1, VHL, VEGFA, UCP2, TP53, TIMP1, TAF9, AURKA, SOX9, SOD2, SOD1, SHC1, BCL2, RELA, OPA1, NGF, NFKB1, MPO, MMP9, SMAD3, LRP1, LEP, KDR, FAS, IL10, IL6ST, IL6, IL4, IL2, IGF1, HSPD1, HSPA5, ANXA5, HIF1A, HGF, HDAC2, GSK3B, GAS6, AKR1B1, ALB, AKT1, EPO, ITCH, DPEP1, GHRL
Positive regulation of gene expression	48	486	4.56741E−41	CDKN2A, CD34, CD28, CASR, TNFSF11, CALCR, FGF23, WT1, VIM, VEGFA, VDR, TP53, TNF, TLR4, TGFB1, SOX9, BMP2, RET, PLAG1, PF4, ATM, NOS3, NGF, MSN, SMAD3, SMAD2, KIT, APP, INS, IL18, IL8, IL6, IL4, IL1B, APOB, IGF1, IFNG, HIF1A, GSN, GSK3B, AMH, GAS6, FN1, AKT1, F3, ENG, EGF, CRP
Response to drug	39	279	5.19875E−39	CDKN1A, ADIPOQ, CAT, CASP3, XRCC1, UMOD, SCGB1A1, TP53, HNF1B, SST, SOD2, SOD1, BGLAP, BCL2, BCHE, RET, REN, RELA, PTH, PPARG, ABCB1, TNFRSF11B, MTHFR, MAS1, LTA, LPL, RHOA, IL10, IGFBP2, APOA1, ICAM1, APEX1, HSPD1, HMOX1, HDAC2, AMH, FABP3, ENG, EDN1
Positive regulation Of transcription by RNA polymerase II	59	1068	7.65307E−37	CDKN2A, CD40, CD28, RUNX2, TP63, TNFSF11, CNBP, WT1, VEGFA, VDR, TP53, TNF, TLR4, TGFB1, HNF1B, TAF9, SOX9, BMP2, RELA, REL, PTH, PPARG, PPARA, POMC, PLAG1, PF4, PER1, SERPINE1, ATM, NOS1, NODAL, NFKB1, MAX, SMAD4, SMAD3, SMAD2, IRF1, APP, IL18, IL17A, IL10, IL6, IL4, IL2, IGF1, APEX1, HNF4A, HIF1A, HGF, HDAC2, AKT1, ESR1, EPAS1, ENG, MIXL1, BCL2L12, EDN1, DDIT3, ADRB2
Inflammatory response	41	403	1.72781E−35	CD44, CD40LG, CD40, CALCA, UMOD, TNFRSF1B, TNF, TLR4, TGFB1, TAC1, SPP1, BMP2, SELP, CCL5, CCL2, RELA, REL, PTGS2, PF4, NFKB1, MEFV, KNG1, KIT, IDO1, IL18, IL17A, IL8RB, IL8, IL6, IL2RA, IL1B, HSPG2, AKT1, AGTR1, ITCH, AGER, DPEP1, F11R, CRP, ADM, CC5
Response to lipopolysaccharide	31	172	2.05775E−34	CASP3, VCAM1, UMOD, SCGB1A1, TNFRSF1B, TLR4, THBD, TFPI, TAC1, SOD2, SELP, REN, RELA, NOS3, NOS1, MPO, LTA, JAK2, FASLG, IDO1, IL10, IL1B, APOB, ICAM1, HSPD1, HDAC2, GGT1, EPO, EDN1, CYP27B1, ADM
Cellular protein metabolic process	32	198	5.1266E−34	CALCA, FGF23, TTR, TIMP1, TF, SPP1, SAA1, PRL, SERPINA1, P4HB, NPPA, MMP2, MMP1, KNG1, APP, INS, APOE, IL6, APOB, IGFBP3, IGFBP2, IGFBP1, IGF1, APOA1, HSPG2, GSN, GAS6, FN1, ALB, AHSG, CST3, CP
Response to hypoxia	30	170	4.716E−33	ADIPOQ, CAT, CASP3, XRCC1, VEGFA, VCAM1, UCP2, BMP2, SOD2, PPARA, ATM, NOS1, MTHFR, MMP2, SMAD4, SMAD3, LTA, RHOA, LEP, ICAM1, HSPD1, HMOX1, HIF1A, EPO, EPAS1, ENG, EDN1, AGER, HIF3A, ADM

To study the function of TMGs and the enrichment of signaling pathways, KEGG pathway enrichment analysis was also performed on the identified TMGs using GeneCodis. The KEGG pathway enrichment analysis identified 242 important pathways involving 270 TMGs. Table 2 shows that most of the important signaling pathways that were enriched were "pathways in cancer" (*P* = 2.40582E−63), "cytokine-cytokine receptor interaction" (*P* = 9.03679E−52), "AGE-RAGE signaling pathways in diabetic complications" (*P* = 1.20609E−43), "PI3K-Akt signaling pathway" (*P* = 1.90881E−39) and "HIF-1 signaling pathway " (*P* = 6.07415E−36) involved 58, 39, 29, 36 and 25 TMGs, respectively (Table 2). Other highly concentrated pathways included the “metabolic pathways”, “proteoglycans in cancer”, “transcriptional misregulation in cancer”, “fluid shear stress and atherosclerosis” and "Human cytomegalovirus infection".

**Table 2:** Top 10 enriched KEGG pathways assigned to the text mining genes

Process	Genes in query set	Total genes in genome	Corrected hypergeometric *P* value	Genes
Pathways in cancer	58	370	2.40582E−63	CDKN2A, CDKN1A, CASP3, FGF23, VHL, VEGFA, TP53, TGFB1, BMP2, BCR, BCLBAX, RET, RELA, PTGS2, PPARG, NOTCH3, NFKB1, MMP9, MMP2, MMP1, MAX, SMAD4, SMAD3, SMAD2, RHOA, KNG1, KIT, JAK2, FASLG, FAS, IL8, IL6ST, IL6, IL4, IL2RA, IL2, IGF1, IFNG, IFNA1, HMOX1, HIF1A, HGF, HDAC2, GSTT1, GSTM1, GSK3B, GRB2, GNB3, FN1, AKT1, ESR1, EPO, EPAS1, AGTR1, AGT, EGF, EDN1
Cytokine-cytokine receptor interaction	39	148	9.03679E−52	CD40LG, CD40, CD4, TNFRSF10A, TNFSF10, TNFSF11, TNFRSF1B, TNF, TGFB1, BMP2, CCL5, CCL2, TNFRSF17, PRL, PF4, TNFRSF11B, NODAL, NGF, LTA, LEP, FASLG, FAS, IL18, IL17A, IL10, IL8RB, IL8, IL6ST, IL6, IL4, IL2RA, IL2, IL1B, IFNG, IFNA1, AMH, GH1, EPO, CCR5
AGE-RAGE signaling pathway in diabetic complications	29	77	1.20609E−43	CASP3, VEGFA, VCAM1, TNF, THBD, TGFB1, CCL2, BCL2, BAX, RELA, SERPINE1, NOS3, NFKB1, MMP2, SMAD4, SMAD3, SMAD2, JAK2, IL8, IL6, IL1B, ICAM1, FN1, AKT1, F3, AGTR1, AGT, EDN1, AGER
PI3K-Akt signaling pathway	36	225	1.90881E−39	CDKN1A, FGF23, YWHAE, VWF, VEGFA, TSC2, TP53, TLR4, SPP1, BCL2, RELA, PRL, NOS3, NGF, NFKB1, KIT, KDR, JAK2, FASLG, IRS1, INS, IL6, IL4, IL2RA, IL2, IGF1, IFNA1, HGF, GSK3B, GRB2, GNB3, GH1, FN1, AKT1, EPO, EGF
HIF-1 signaling pathway	25	76	6.07415E−36	CDKN1A, VHL, VEGFA, TLR4, TIMP1, TF, BCL2, RELA, EPO, SERPINE1, NPPA, NOS3, NFKB1, INS, IL6, IGF, IFNG, HMOX1, HIF1A, GAPDH, AKT1, ENO2, ENO1, EGF, EDN1
Metabolic pathways	44	559	7.22668E−35	PHGDH, CEL, HPSE, KL, CAT, TKTL1, UROD, TYRP1, SCD, SAT1, ACSM3, RENBP, PTGS2, PIK3C2A, PAH, NOS3, NOS1, NEU1, NAGLU, MTHFR, ARG2, LBR, IDO1, HSD11B1, HMOX1, ACACA, GSTT1, GSTM1, GSR, GLA, GGT1, GAPDH, FUT2, AKR1B1, ALDH2, ENO2, ENO1, CNDP1, DPYD, CYP27B1, CYP3A5, CHDH, CYP3A4, COMT
Proteoglycans in cancer	27	142	9.0523E−32	HPSE, CDKN1A, CD63, CD44, CASP3, VEGFA, TP53, TNF, TLR4, TGFB1, MSN, MMP9, MMP2, SMAD2, RHOA, KDR, FASLG, FAS, IGF1, HSPG2, HIF1A, HGF, GRB2, FN1, FLNB, AKT1, ESR1
Transcriptional misregulation in cancer	25	112	2.446E−31	CDKN1A, CD40, RUNX2, WT1, TP53, BAX, RELA, REL, PPARG, PLAT, ATM, NFKB1, MPO, MMP9, MMP3, MAX, ITGAM, IL8, IL6, IGFBP3, IGF1, HDAC2, GZMB, EYA1, DDIT3
Fluid shear stress and atherosclerosis	23	98	2.08996E−29	VEGFA, VCAM1, TP53, TNF, THBD, CCL2, BCL2, RELA, PLAT, NOS3, NFKB1, MMP9, MMP2, RHOA, KDR, IL1B, IFNG, ICAM1, HMOX1, GSTT1, GSTM1, AKT1, EDN1
Human cytomegalovirus infection	26	161	8.37841E−29	CDKN2A, CDKN1A, CASP3, VEGFA, TSC2, TP53, TNF, CCL5, CCL2, BAX, RELA, PTGS2, NFKB1, RHOA, FASLG, FAS, IL8RB, IL8, IL6, IL1B, IFNA1, GSK3B, GRB2, GNB3, AKT1, CCR5

### PPI network construction, identification of Hub genes and Modular analysis

**Fig. 3:**
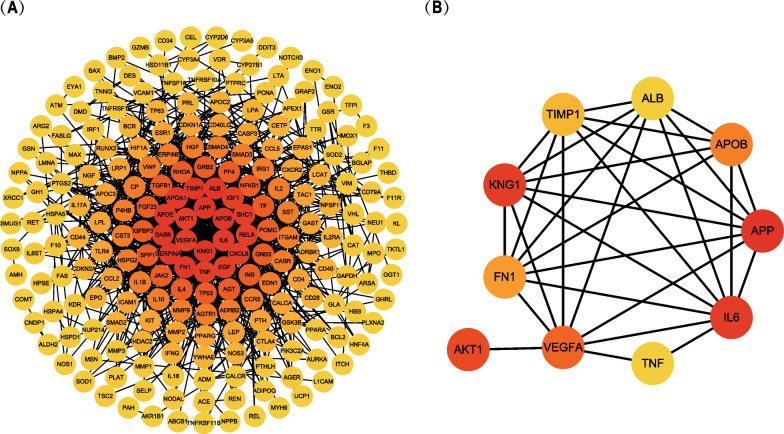
Categorize the degree and analyze Hub genes. **A** All nodes of PPI were presented according to degree by CytoHubba. The degree decreases from inside to outside and the color changes from dark to light. **B** The first 10 hub genes in the macro module were identified by CytoHubba plug-in. The image shows degree from red to yellow, the significance of genes declines

**Fig. 4:**
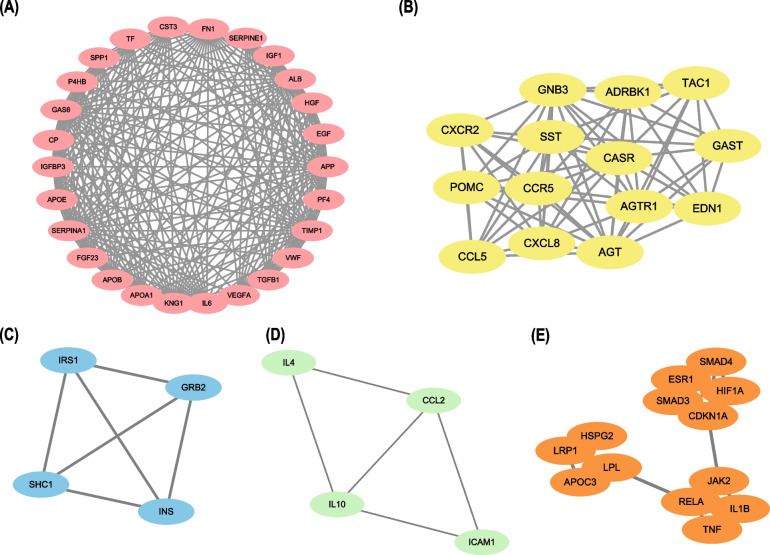
Description and enrichment analysis of the TMGs. **A–E** The five modules were carried out from PPI network using MCODE. **A** Module 1, the most significant module with 26 nodes; **B** Module 2; **C** Module 3; **D** Module 4; **E** Module 5

Various biological data networks can be obtained through correlation analysis, including information about signal transduction pathways, gene regulation and protein–protein interaction. We input the target genes into the STRING website. A PPI network of 270 target genes was constructed through analysis. The constructed PPI network consisted of 216 nodes and 1087 edges (Fig. 2B). Five of those genes were not included in the constructed PPI network. We ran the plugin CytoHubba in Cytoscape. The topological network algorithm was used to assign a value to each gene and rank each gene according to its degree in the correlation analysis result. The darker the color, the higher the score and the more significant the gene (Fig. 3A). According to the analysis results, 79 nodal genes with nodal degree ≥ 10 were selected (Table 3). Then, we found the hub genes in the PPI network, which referred to the gene with the highest connection in the module. As illustrated in Fig. 3B, *APP*, *IL6*, *KNG1*, *AKT1*, *VEGFA*, *APOB*, *FN1*, *TIMP1*, *ALB* and *TNF* were the 10 hub genes with the highest connectivity in the module. If these selected genes were supported by relevant clinical research data in the research progress, we could further study the association between these proteins and patient survival. To further obtain more gene modules, we used the MCODE plugin to analyze the 79 target genes screened in the previous step and obtained 5 modules (Fig. 4A–E). We selected the two modules with the highest scores (including 40 genes) by calculating network scores for further analysis. Module 1 contained 26 nodes and 241 edges. Module 2 contained 14 nodes and 69 edges.

**Table 3:** Hub node genes in the PPI network identified with filtering node degree ≥ 10

Name	Degree	MCC	Name	Degree	MCC
APP	50	9.22E+13	P4HB	19	9.22E+13
IL6	44	9.22E+13	CP	19	9.22E+13
KNG1	44	9.22E+13	HSPG2	18	870
AKT1	38	1.86E+03	SMAD4	18	134
VEGFA	35	8.72E+10	ITGAM	18	25
APOB	33	9.22E+13	IL1B	17	6744
FN1	31	9.22E+13	VWF	17	8.72E+10
TIMP1	30	9.22E+13	HGF	17	8.72E+10
ALB	29	9.22E+13	IL2	17	5088
TNF	29	6252	CASR	17	4.04E+07
APOA1	28	9.22E+13	CCR5	17	3.99E+07
EGF	28	8.72E+10	ADRB2	17	6056
SHC1	26	4286	SMAD3	16	128
RELA	26	3075	IRS1	16	1634
CXCL8	26	4.00E+07	MMP9	15	104
SERPINA1	25	9.22E+13	SST	15	4.00E+07
GAS6	25	9.22E+13	CCL5	14	3.99E+07
APOE	25	9.22E+13	HIF1A	13	99
IGF1	25	8.72E+10	LEP	13	95
GRB2	25	4154	CCL2	12	2916
INS	25	2607	LPL	12	2169
TP53	25	85	LRP1	12	1474
GNB3	24	4.04E+07	ESR1	12	48
JAK2	23	2276	ADRBK1	12	378,240
TGFB1	23	8.72E+10	PTH	12	5050
RHOA	23	946	CD40LG	11	72
PF4	23	8.72E+10	CASP3	11	30
SPP1	22	9.22E+13	CXCR2	11	3.99E+07
IGFBP3	22	9.22E+13	TAC1	11	403,206
NFKB1	22	880	CD4	11	729
TF	22	9.22E+13	PPARG	11	43
AGT	22	4.04E+07	MMP2	10	66
IL4	21	6585	KIT	10	771
FGF23	21	9.22E+13	ICAM1	10	731
POMC	21	4.00E+07	TLR4	10	844
CST3	20	9.22E+13	APOC3	10	4320
SERPINE1	20	8.72E+10	CDKN1A	10	75
EDN1	20	368,215	GAST	10	403,200
AGTR1	20	449,598	CALCA	10	5.05E+03
IL10	19	3815			

Degree: Represents the number of connections between a node and other nodes. In network analysis, the higher the degree of a protein, the correlation between it and many other proteins is proved and it can be considered as a key protein

MCC (Maximal Clique Centrality): MCC algorithm can calculate the core targets in the network and has been proved to be an accurate method for predicting important targets in CytoHubba

**Fig. 5:**
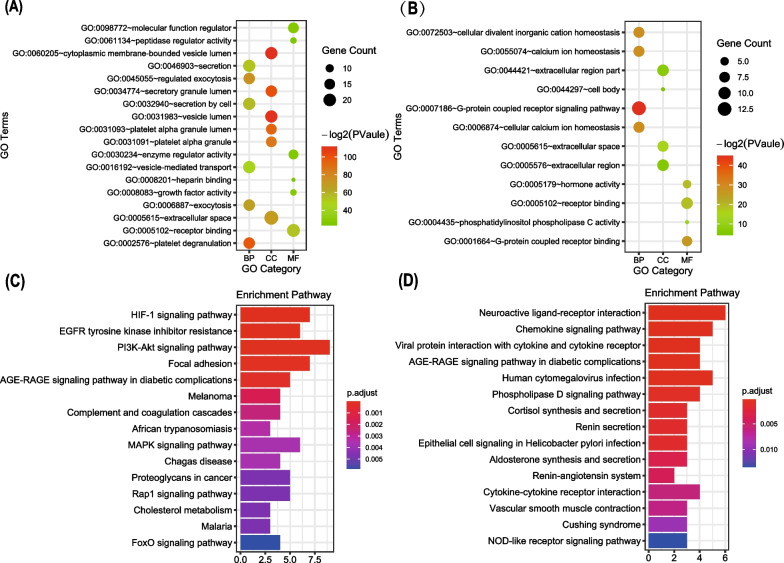
Gene ontology and KEGG pathway analysis of the genes in the first two modules. **A** Top 18 significantly enriched GO terms in module 1. **B** Top 12 Significantly enriched GO terms in module 2. **C** Top 15 significantly enriched KEGG pathways in module 1. **D** Top 15 significantly enriched KEGG pathways in module 2. The functional and pathway enrichment analyses were performed using DAVID. KEGG: Kyoto Encyclopedia of Genes and Genomes

**Fig. 6:**
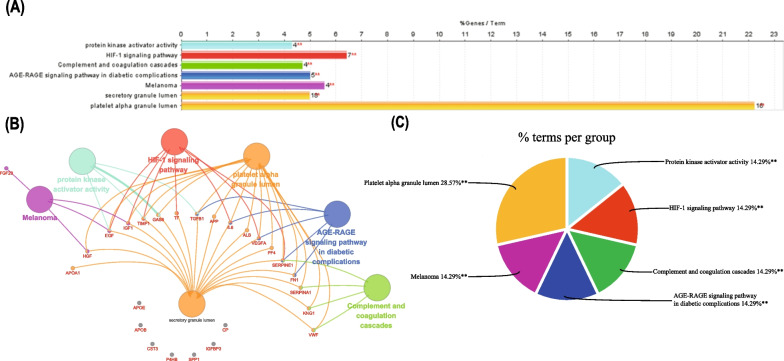
Function analysis of the 26 core genes in module 1. **A** Enriched GO terms and KEGG pathways. **B** Functions and pathways of the core genes were computed and visualized using ClueGO. **C** Distribution of the functions and pathways among the core genes. Each function or pathway is color coded. Corrected *P* < 0.01 was considered statistically significant. KEGG: Kyoto Encyclopedia of Genes and Genomes

In order to further analyze the enrichment of core genes, we conducted Gene ontology and KEGG pathway analysis for the two modules selected in the previous step. The results showed that the 26 genes in module 1 were mainly related to platelet degranulation (BP), cytoplasmic membrane-bounded vesicle lumen (CC) and receptor binding (MF) (Fig. 5A). On the other hand, the 14 genes in module 2 were mainly associated with G-protein coupled receptor signaling pathway (BP), extracellular space (CC), and G-protein coupled receptor binding (MF) (Fig. 5B). Pathway enrichment analysis showed that the genes in module 1 were associated with the HIF-1, PI3K-Akt, MAPK, Rap1, and FoxO signaling pathways (Fig. 5C). Conversely, the genes in module 2 were significantly correlated with the chemokine, phospholipase, and Nod-like receptor signaling pathways (Fig. 5D). Module 1 contained 26 genes with 241 edges, all of which were core genes, indicating that module 1 played a vital role in the PPI network. 26 genes were selected as core candidate genes for the PPI networks. The enrichment analysis indicated that these genes were significantly enriched in platelet alpha granule lumen, HIF-1 signaling pathway, Melanoma, protein kinase activator activity, Complement and coagulation cascades and AGE-RAGE signaling pathway in diabetic complications (*P* < 0.01, Fig. 6).

### Drug-gene interaction analysis of core genes

**Fig. 7:**
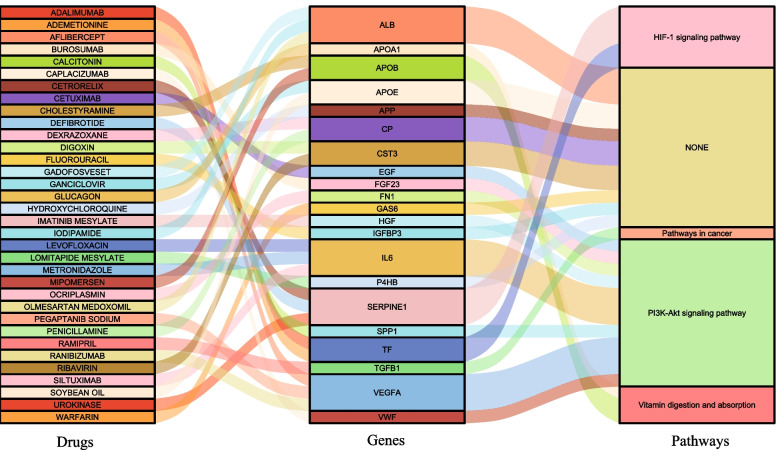
Sankey diagram display the essential connections among drugs, genes, and pathways. Drug-gene interactions were analyzed for 26 genes in module 1. We found 21 genes that target 34 potential existing drugs. In addition, these 21 genes are mainly enriched in 4 KEGG pathways. "None" in the pathway means that the core gene has no related pathway

The final confirmed genes were used to conduct drug-gene interaction analysis and an initial list of 34 drugs was obtained (Table 4). Thirty-four drugs targeted by 21 of the 26 core genes (except *IGF1*, *KNG1*, *PF4*, *SERPINA1* and *TIMP1*) may be potential therapeutic agents for renal insufficiency in geriatric multimorbidity patients. The 26 core genes in the analysis results were mainly enriched in PI3K-Akt signaling Pathway, HIF-1 signaling Pathway, Pathways in cancer and Vitamin digestion and absorption. The main links between drugs, genes, and pathways are displayed in Fig. 7.

**Table 4:** Details of the 34 drugs that potentially target of the 26 core genes

Number	Drug	Genes	Interaction	Score	Drug class	Approved?	PubMed ID
1	BUROSUMAB	FGF23	antagonist	255.17	Not available	NO	29545670
2	ADEMETIONINE	TF	N/A	15.95	Not available	NO	None found
3	ADALIMUMAB	TF	N/A	3.75	Not available	Yes*	27115882
4	CAPLACIZUMAB	VWF	inhibitor	13.67	Antibody fragment	NO	None found
5	SILTUXIMAB	IL6	antagonist	10.21	Therapeutic antibodies	Yes*	8823310
6	LEVOFLOXACIN	IL6	N/A	1.28	Not available	NO	12714806
7	METRONIDAZOLE	IL6	N/A	1.28	Not available	NO	12111578
8	RANIBIZUMAB	VEGFA	inhibitor	8.81	Antibody fragment	Yes*	18046235
9	PEGAPTANIB SODIUM	VEGFA	antagonist	3.36	Aptamer	Yes*	23953100
10	AFLIBERCEPT	VEGFA	antibody	2.35	Therapeutic antibodies/fusion protein	NO	22813448
11	LOMITAPIDE MESYLATE	P4HB	inhibitor	3.54	Not available	Yes*	None found
12	CALCITONIN	SPP1	N/A	3.54	Small molecule	Yes*	8013390
13	CETUXIMAB	EGF	N/A	3.38	Therapeutic antibodies	Yes*	25677871
14	DEFIBROTIDE	SERPINE1	N/A	3.19	Not available	Yes*	12745658
15	UROKINASE	SERPINE1	inducer	3.19	Thrombolytic agents/protein	Yes*	12709915
16	CETRORELIX	SERPINE1	N/A	2.13	Fertility agents/peptide	Yes*	16391860
17	GADOFOSVESET	ALB	N/A	3.04	Not available	NO	None found
18	IODIPAMIDE	ALB	N/A	3.04	Not available	NO	None found
19	OLMESARTAN MEDOXOMIL	ALB	N/A	3.04	Not available	NO	22086979
20	CHOLESTYRAMINE	APOB	N/A	2.36	Not available	Yes*	3906004
21	MIPOMERSEN	APOB	N/A	1.77	Antisense oligo	Yes*	None found
22	RIBAVIRIN	CST3	N/A	1.93	Not available	Yes*	18637076
23	DIGOXIN	CST3	N/A	1.88	Cardiotonic agents/small Molecule	Yes*	17698593
24	GANCICLOVIR	APOE	N/A	1.88	Not available	Yes*	16322528
25	SOYBEAN OIL	APOE	N/A	1.5	Not available	Yes*	3021887
26	WARFARIN	GAS6	N/A	1.77	Anticoagulants/small molecule	Yes*	16014032
27	GLUCAGON	APOA1	N/A	1.59	Not available	Yes*	3130065
28	DEXRAZOXANE	CP	N/A	1.52	Not available	Yes*	8285144
29	PENICILLAMINE	CP	N/A	1.3	Not available	Yes*	11721763
30	OCRIPLASMIN	FN1	cleavage	1.29	Not available	Yes*	23193358
31	IMATINIB MESYLATE	HGF	N/A	0.91	Not available	NO	11439348
32	RAMIPRIL	TGFB1	N/A	0.86	Antihypertensive agents	Yes*	15716710
33	FLUOROURACIL	IGFBP3	N/A	0.61	Not available	Yes*	20860465
34	HYDROXYCHLOROQUINE	APP	N/A	0.37	Small molecule	Yes*	11117548

Interaction: The nature of drug interaction with target genes

Score: Drug interaction scores with target genes

Approved?: (Yes*) Drugs that have been approved by the US Food and Drug Administration

## Discussion

By applying a series of bioinformatics methods to explore gene expression, we identified 351 genes involved in renal insufficiency in geriatric multimorbidity patients. Among these genes enriched go biological process terms, which have so far been shown to be primarily associated with renal insufficiency iucluding cytokine-mediated signaling pathway [30], signal transduction [31], negative regulation of apoptotic process [32], positive regulation of gene expression [33], response to drugs [34], positive regulation Of transcription by RNA polymerase II [35], inflammatory response [36], response to lipopolysaccharide [37], cellular protein metabolic process [38] and response to hypoxia [39]. In addition, the results of KEGG pathway analysis corresponding to 351 genes related to renal insufficiency including cytokine-cytokine receptor interaction [40], AGE-RAGE signaling pathways in diabetic complications [41], PI3K-Akt signaling pathway [42], HIF-1 signaling pathway [42], metabolic pathways [43], proteoglycans in cancer [44], transcriptional misregulation in cancer [45], fluid shear stress and atherosclerosis [46] and Human cytomegalovirus infection [47]. Pathways in cancer may be associated with renal impairment, but the correlation needs to be further verified.

The PPI network and CytoHubba explored the correlation connectivity between these genes (Fig. 3A) and identified 10 pivotal genes, including *APP*, *IL6*, *KNG1*, *AKT1*, *VEGFA*, *APOB*, *FN1*, *TIMP1*, *ALB,* and *TNF*(Fig. 3B). Relevant literature have shown that *IL6*, *AKT1*, *VEGFA*, *FN1*, *TIMP1*, *ALB* and *TNF* are associated with renal insufficiency. According to current literature, there are no studies on the other hub genes and their effect on renal insufficiency. However, these genes may also play a significant role in renal insufficiency. The MCODE analysis generated 26 core genes, including *CST3*, *SERPINA1*, *FN1*, *PF4*, *IGF1*, *KNG1*, *IL6*, *VEGFA*, *ALB*, *TIMP1*, *TGFB1*, *HGF*, *SERPINE1*, *APOA1*, *APOB*, *FGF23*, *EGF*, *APOE*, *VWF*, *TF*, *CP*, *GAS6*, *APP*, *IGFBP3*, *P4HB, * and *SPP1*, that participated in the HIF-1, PI3K-Akt, MAPK, Rap, and FoxO signaling pathways.

The results showed that Interleukin 6 (*IL-6*) is an autocrine growth factor secreted by mesangial cells and is involved in the pathological proliferation of mesangial cells [48]. A meta-analysis showed that serum cystatin C is superior to serum creatinine as a marker of renal function [49]. Serum albumin levels have been shown to further predict the clinical outcomes in patients with CKD undergoing cardiac resynchronization therapy [50]. Among these genes, *APOA1*, *APOB*, and *APOE* all belong to the apolipoprotein family. ApoA1 was negatively associated with eGFR decline during a short period of one year [51]. APOB-containing lipoproteins complex of the medium and low-density apolipoprotein complexes may contribute to renal insufficiency by interacting with glomerular or tubulointerstitial problems [52]. Patients with high *APOE2* levels have a higher risk of developing CKD or even ESRD because *APOE2* affects the clearance of very-low-density lipoprotein (VLDL) and chylomicron (CM) remnants [53]. *FN1* mutations lead to glomerular disease with fibronectin deposition [54]. Excessive *FN* production can accelerate glomerulosclerosis and tubulointerstitial fibrosis and increase the incidence of diabetic nephropathy (DN) by causing the thickening of the glomerular and tubular basement membranes [55]. Fibroblast growth factor 23 (*FGF23*) regulates phosphate reabsorption and 1alpha-hydroxylase activity in the kidney [56]. Studies have shown that *FGF23* levels appear to be independently associated with mortality in dialysis patients [57]. *SPP1* (Osteopontin) is a cell-attached glycoprotein and its expression correlated with the severity of the renal tubulointerstitial injury [58]. In the process of renal aging, the high expression of *TIMP-1* up-regulates the expression of *PTEN* through an MMP-independent pathway and subsequently leads to aging-related vascular damage [59]. *TGFB1* can further induce renal interstitial fibrosis through endothelial-to-mesenchymal transition (*EndMT*) [60]. Individuals with renal insufficiency with increased *VWF* levels may have an increased risk of venous thrombosis [61]. Decreased vascular endothelial growth factor A (*VEGFA*) levels are associated with glomerular microangiopathy [62]. Currently, there are few studies on the direct relationship between *APP*, *CP*, *EGF*, *GAS6*, *HGF*, *IGF1*, *IGFBP3*, *KNG1*, *SERPINE1*, *SERPINA1*, *TF*, *PF4* and *P4HB*, and renal insufficiency in relevant literature, which can provide a reference for future studies on renal insufficiency with geriatric multimorbidity patients.

HIF is involved in the renal fibrosis process during the disease course of CKD through gene transcription, signaling pathways, epithelial-mesenchymal transition and epigenetic regulation [63]. The PI3K-Akt signaling pathway is mainly involved in regulating cell proliferation, migration, differentiation and angiogenesis [64]. Helix B surface peptide (*HBSP*) can improve renal ischemia–reperfusion injury, renal function and also improve apoptosis after ischemia–reperfusion injury by regulating the PI3k/Akt pathway [65]. Activating mitogen-activated protein kinase (*MAPK*) and lipopolysaccharide (*LPS*) can lead to the increased transcription of pro-inflammatory cytokines, which can directly affect renal parenchyma, promote renal tubular cell apoptosis and directly induce AKI [66]. Decreased *RAP1-GTP* mediated by elevated *RAP1GAP* levels may be a crucial factor in inducing podocyte dysfunction in human glomerular disease [67]. FoxO bound to β-catenin can prevent rHTGF-1-induced profibrosis [68].

As many drugs are metabolized through the kidneys, they might damage renal function. Potential drugs identified through the search of drug-gene interaction are mainly divided into protecting renal function, damaging renal function and adjusting doses according to renal function. Burosumab treats x-linked patients with hypophosphatemia (*XLH*) by antagonizing *FGF23*, increasing renal tubular phosphate reabsorption and normalizing serum phosphorus concentrations [69]. Additional preclinical and clinical studies are warranted to determine whether blocking *FGF23* with burosumab will provide a new targeted intervention for mineral metabolism disorders in CKD [70]. Adalimumab (ADA) is a tumor necrosis factor (*TNF-α*) inhibitor that reduces or suppresses inflammatory processes by inhibiting pro-inflammatory cytokines [71]. ADA pretreatment may play a role in experimental renal insufficiency [72]. Siltuximab inhibited tumor growth of human renal carcinoma in nude mice by binding to *IL-6* [73]. Related studies have shown that angiotensin receptor blockers (ARB) can play a role in renal protection through multiple and complex mechanisms [74]. Olmesartan may delay or prevent microalbuminuria in type 2 diabetes mice, improving renal function [75]. In animal models, ramipril significantly reduced the number of glomerular and tubule-interstitial fibrosis and activated fibroblasts, exerting a protective effect on the kidney [76]. Defibrotide can prevent the upregulation of endothelial dysfunction markers induced by a uremic environment, and downregulate the expression of *HDACs* through the PI3/AKT signaling pathway, thus playing a protective role in the endothelia [77]. In addition, cholestyramine [78], mipomersen [79] and soybean oil can regulate lipid metabolism and directly improve renal function [80]. Glp-1 RA inhibits NHE3-dependent sodium reabsorption in the proximal tubule and has a direct renal protective effect on the renin-angiotensin system, which can improve inflammation, ischemia/hypoxia, apoptosis and neural signaling [81]. Studies have shown that imatinib mesylate interferes with *PDGF* and *TGF-β* activated signaling cascade and improves renal tubulointerstitial fibrosis [82]. Hydroxychloroquine (HCQ) reduces renal insufficiency by downregulating *NLRP3* inflammasomes activation mediated by *CTSB* and *CTSL* [83].

According to literature, ranibizuma [84], cetuximab [85], aflibercept [85], warfarin [86], gadofosveset [87], fluorouracil [88] and penicillamine [89] may cause renal insufficiency through a variety of ways. Moreover, the dose of ribavirin [90], ganciclovir [91], metronidazole [92], levofloxacin [93] and digoxin [94] needs to be adjusted according to renal function. Currently, the relationship between ademetionine, caplacizumab, pegaptanib sodium, lomitapide mesylate, calcitonin, urokinase, cetrorelix, iodipamide, dexrazoxane, ocriplasmin, and renal insufficiency has not been reported in the existing literature. Future studies could further investigate the association between these drugs and renal insufficiency.

In conclusion, we identified 26 core genes, *CST3*, *SERPINA1*, *FN1*, *PF4*, *IGF1*, *KNG1*, *IL6*, *VEGFA*, *ALB*, *TIMP1*, *TGFB1*, *HGF*, *SERPINE1*, *APOA1*, *APOB*, *FGF23*, *EGF*, *APOE*, *VWF*, *TF*, *CP*, *GAS6*, *APP*, *IGFBP3*, *P4HB,* and *SPP1*, that may be related to renal insufficiency in patients with geriatric multimorbidity. These genes were enriched in the HIF-1, PI3K-Akt, MAPK, Rap1, and FoxO signaling pathways. We also identified 34 drugs that may help guide the future treatment of renal insufficiency in patients with geriatric multimorbidity. The lack of experimental verification is a limitation of this study, and further experimental studies are needed to verify these results.

## Data Availability

The authors declare that the data supporting the findings of this study are available within the article. The datasets analysed during the current study are publicly available in the pubmed2ensembl (http://www.pubmed2ensembl.org).

## Abbreviations

GO: Gene Ontology
KEGG: Kyoto Encyclopedia of Genes and Genomes
MCODE: Molecular Complex Detection
DAVID: Database for Annotation, Visualization and Integrated Discover
CKD: Chronic kidney disease
eGFR: Estimating glomerular filtration rate
KDIGO: Kidney Disease: Improving Global Outcomes
CKD-EPI: Chronic Kidney Disease Epidemiology Collaboration
ESRD: End-stage renal disease
PPI: Protein–protein interaction
DGIDB: Drug-gene interaction database
VLDL: Very-low-density lipoprotein
CM: Chylomicron
DN: Diabetic nephropathy
FGF23: Fibroblast growth factor 23
EndMT: Endothelial-to-mesenchymal transition
VEGFA: Vascular endothelial growth factor A
